# Pinpointing the vesper bat transposon revolution using the *Miniopterus natalensis* genome

**DOI:** 10.1186/s13100-016-0071-y

**Published:** 2016-07-22

**Authors:** Roy N. Platt, Sarah F. Mangum, David A. Ray

**Affiliations:** Department of Biological Sciences, Texas Tech University, Box 43131, Lubbock, TX 79409-3131 USA

**Keywords:** Miniopteridae, Vespertilionidae, TcMariner, Helitron, Transposable element

## Abstract

**Background:**

Around 40 million years ago DNA transposons began accumulating in an ancestor of bats in the family Vespertilionidae. Since that time, Class II transposons have been continuously reinvading and accumulating in vespertilionid genomes at a rate that is unprecedented in mammals. *Miniopterus* (Miniopteridae), a genus of long-fingered bats that was recently elevated from Vespertilionidae, is the sister taxon to the vespertilionids and is often used as an outgroup when studying transposable elements in vesper bats. Previous wet-lab techniques failed to identify *Helitrons*, TcMariners, or hAT transposons in *Miniopterus*. Limitations of those methods and ambiguous results regarding the distribution of piggyBac transposons left some questions as to the distribution of Class II elements in this group. The recent release of the *Miniopterus natalensis* genome allows for transposable element discovery with a higher degree of precision.

**Results:**

Here we analyze the transposable element content of *M. natalensis* to pinpoint with greater accuracy the taxonomic distribution of Class II transposable elements in bats. These efforts demonstrate that, compared to the vespertilionids, Class II TEs are highly mutated and comprise only a small portion of the *M. natalensis* genome. Despite the limited Class II content, *M. natalensis* possesses a limited number of lineage-specific, low copy number piggyBacs and shares several TcMariner families with vespertilionid bats. Multiple efforts to identify *Helitrons,* one of the major TE components of vesper bat genomes, using de novo repeat identification and structural based searches failed.

**Conclusions:**

These observations combined with previous results inform our understanding of the events leading to the unique Class II element acquisition that characterizes vespertilionids. While it appears that a small number of TcMariner and piggyBac elements were deposited in the ancestral *Miniopterus* + vespertilionid genome, these elements are not present in *M. natalensis* genome at high copy number. Instead, this work indicates that the vesper bats alone experienced the expansion of TEs ranging from *Helitrons* to piggyBacs to hATs.

**Electronic supplementary material:**

The online version of this article (doi:10.1186/s13100-016-0071-y) contains supplementary material, which is available to authorized users.

## Background

Transposable elements (TEs) are genetic elements with the ability to mobilize throughout a host genome. Often TE copies are generated as a result of the mobilization process and TEs can end up occupying large portions of mammalian genomes. For example, between 45 and 70 % of the human genome is occupied by TEs [1, 2]. TEs are classified into two major classes based on their mobilization mechanism. Class I elements, also known as retrotransposons, mobilize as an RNA intermediate that is reverse transcribed back into the genome. These elements are referred to as "copy and paste" elements since they generate identical copies of themselves upon insertion. Retrotransposons are further classified into Long Terminal Repeats (LTRs), Long INterspersed Elements (LINEs), and Short INterspersed Elements (SINEs). Class II elements, also known as DNA transposons, mobilize via a transposase enzyme. During mobilization, the terminal inverted repeat-containing DNA transposons physically excise from the genome and re-integrate at another locus. However, in addition to these canonical "cut and paste" DNA transposons, *Helitrons* and Mavericks mobilize through other mechanisms that do not fully excise the template TE. As a result, these Class II elements are "copy and paste" transposons since they mobilize through a single DNA strand excised from the parent locus.

In general, retrotransposons are much more common in mammalian genomes than DNA transposons. For example, 43 % of the human genome is derived from retrotransposons vs. 3 % from DNA transposons [2]. In addition to being less frequent, transposons are often found in genomes as heavily mutated insertions; indicating long periods of inactivity. The single major exception to this general trend is the presence of recently inserted Class II elements in the genomes of vespertilionid bats [3, 4]. As much as 6 % of the *Myotis lucifugus* genome is derived from recently active *Helitrons* [5], ~3.5 % from cut and paste transposons [6], and half of all recent TE accumulation appears to come from Class II elements [7].

To understand the timing and evolutionary implications of this unique activity, we must first identify the taxonomic distribution and accumulation patterns of the elements involved. Previous work focusing on the initial horizontal transfer or reactivation of Class II elements in vespertilionids indicated that *Helitrons* are restricted to the vespertilionid lineage [8] and only a limited number of cut and paste transposon families are found beyond Vespertilionidae [6]. These results were based on comparisons of vespertilionids to several non-vesper bats including *Miniopterus*, a genus of long-fingered bats recently elevated to familial level from Vespertilionidae [9]. For example, using internal PCR primers, Ray et al. [6] tried to amplify piggyBac, hAT, and TcMariner elements in a panel of chiropteran including *Artibeus jamaicensis*, *Balionycteris* sp., *Corynorhinus rafinesquii*, *Eptesicus furinalis*, *Hipposideros cervinus*, *Kerivoula papillosa*, *Macroglossus sobrinus*, *Miniopterus* sp., *Myotis austroriparius*, *My. horsfieldii*, *Natalus stramineus*, *Nycticeius humeralis*, *Pteronotus parnellii*, *Rhinolophus borneoensis*, and *Thyroptera tricolor*. Results indicated that TcMariner elements were only present in vespertilionids (*C. rafinesquii*, *E. furinalis*, *K. papillosa*, *Myotis austroriparius*, *Myotis horsfieldii*, and *N. humeralis*). hATs and piggyBacs were only found in *Myotis* species, with the exception of one piggyBac (piggyBac2_ML) that was amplified in *Myotis* sps. and *Miniopterus* but was absent in other all other samples including the non-*Myotis* vespertilionids [6]. Probe-based hybridization failed to identify *Helitrons* in *Miniopterus* or any other non-vesper bats [8].

Modern genome assembly and sequencing techniques provide many advantages for TE discovery over wet-lab based techniques. Mispriming, in the case of PCR, or reduced hybridization efficiency, in the probe-based analyses, could easily allow elements to be missed in any or all of these genomes. In addition, these methods rely on a priori knowledge of TE content in order to build primers/probes for loci of interest. The recent release of the *Miniopterus natalensis* genome [10] allows these questions to be answered more precisely and with independent and unbiased data. Here, we characterize the repetitive portion of the *M. natalensis* genome with an emphasis on Class II elements in order to understand the acquisition of these Class II TEs in bats.

## Methods

Repeats were identified in the *Miniopterus natalensis* genome using de novo methods and TEs were fully validated [11] as detailed below. Putative repeats were identified using RepeatModeler [12] and the current *M. natalensis* assembly (Genbank accession GCA_001595765.1). The RepeatModeler repeats were masked with RepeatMasker [13] using all known Chiropteran TEs (-species “Chiroptera”) to remove repeats that have already been described in other bat species. Those repeats that were ≥80 % similar to known elements across more than 50 % of their length were excluded from downstream analyses. The remaining elements were considered possible *Miniopterus*-specific elements. To manually validate these repeats, they were used as BLASTn v2.2.27 [14] queries against the *M. natalensis* genome. BLASTn hits were restricted to those with E values greater than 1*e*-10. For each repeat, the forty loci most similar to the BLASTn query were extracted from the genome along with 500 or more bases of flanking sequence and aligned using MUSCLE v3.8.1551 [15]. Repeats with less than 10 BLASTn hits were culled from further analysis. For the remaining repeats, majority-rule consensus sequences were generated for each alignment using BioEdit v7.2.5 [16]. Elements that contained single copy DNA on both the 5' and 3' end were considered to be complete. If an alignment ended within a repetitive portion, the consensus sequence was generated across the entire repetitive portion of the alignment and this new consensus sequence was used as a query in subsequent BLASTn rounds. This process was iterated until all de novo repeats were fully represented.

Beyond RepeatModeler searches, attempts were made to identify low copy number and highly divergent *Helitrons* using HelitronScanner [17]. HelitronScanner searches the genome for 5’ and 3’ terminal sequences associated with *Helitrons*. Terminal sequences are then paired with their closest partner. Those falling within a set distance are considered putative *Helitrons*. Default parameters were used in HelitronScanner searches except for the scoring threshold, which was raised from a default of 5 to 10. As a control, a copy of the *M. natalensis* genome was shuffled using EMBOSS’s shuffleseq (v6.6.0 [18]), and run in parallel using the same parameters. A series of BLAT [19], and BLAST searches were used to validate putative *Helitrons* that resulted from HelitronScanner queries.

All novel repeats were classified based on structural hallmarks (ex. poly-A tails, target site duplications, terminal inverted repeats, etc.) and homology to other TEs present in RepBase (accessed 1 April 2016 [20]). For larger elements, intact open reading frames (ORFs) were identified with ORF Finder [21]. Elements were classified using the 80-80-80 rule [22] and designated based on standard naming conventions implemented by RepBase [20]. For example, two SINEs in *M. natalensis* meet the 80-80-80 thresholds when compared to the canonical VES SINE, but each varies from one another by 5 % at the nucleotide level and contain diagnostic indels. In this case, both SINEs are recognized as members of the separate subfamilies of VES: VES-1_MNa and VES-2_MNa. After classification, the *M. natalensis* repeats were combined with all known mammal TEs from RepBase and used as a customized library to annotate the *M. natalensis* genome. For comparative purposes genomes from closely related bat species, were analyzed using identical RepeatMasker settings to provide a better estimate of the TE dynamics during the *Miniopterus* and Vespertilionidae divergence. These taxa include *Myotis lucifugus* (GCA_000147115.1), *Eptesicus fuscus* (GCA_000308155.1) and *Pteronotus parnellii* (GCA_000147115.1) and were chosen based on their phylogenetic relationships. Repeat accumulation profiles for all taxa were generated using the Kimura 2-parameter distance [23] between the RepeatMasker library and homologous loci in the genome. Highly mutable CpG sites [24] were excluded from distance calculations. Elements belonging to the same superfamily were binned based on their genetic distances. Distances were rounded down to the nearest full percentage. For comparison, average genetic distances between genomic TEs and the consensus library TE were calculated for all DNA transposons occupying more than 10 Kb of any bat genome.

To identify TEs specific to *M. natalensis*, repeats identified by RepeatModeler and successfully validated, were used as BLASTn queries against all other genomes in the NCBI Genomes (chromosomes) database. *M. natalensis* was excluded (NCBI Taxa ID 9432) from these searches. The most closely related species to *M. natalensis* in the NCBI Genome database are the vespertilionids, *Myotis lucifugus*, *Myotis brandtii*, *Myotis davidii*, and *Eptesicus fuscus. Pteronotus parnellii* (family Mormoopidae), serves as an outgroup to a monophyletic clade comprising Vespertilionidae + Miniopteridae [25]. Repeats were classified based on the species distribution of the 50 best BLASTn hits. If the best hits for a repeat belonged to a vespertilionid or *P. parnellii*, the *M. natalensis* repeat was assumed to have been active in the common ancestor of these taxa. If, however, the best hits were to species other than a vespertilionid or *P. parnellii*, then the TE has a distribution among species that does not follow the species tree. If no hits were found to other species, it was assumed that these elements are only found in *M. natalensis* and are lineage-specific. BLASTn hits were only considered if they had an E value greater than 1*e*-10 and were more than 80 % similar across 80 % of the length of the *M. natalensis* query.

## Results

RepeatModeler analysis of the unmasked *Miniopterus natalensis* genome identified 396 putative repetitive sequences. After removing elements with homology to known chiropteran TEs, simple repeats, and low copy number elements 52 putative TEs remained. Of these, 13 were so heavily mutated in the *M. natalensis* genome that generating a consensus sequence was not feasible. The remaining 39 elements were fully validated and classified. In all: 10 LTRs, 2 SINEs, 2 LINEs, and 25 DNA transposons were identified. All LTR elements were solo LTRs of less 1,100 bp. These LTRs were classified as ERV1 (gammaretroviruses) or ERV3s (spumaviruses) based on the size of their target site duplicates. The two SINEs were variants of the VES family of SINEs common in many bats [26, 27]. The two LINEs belonged to the LINE-1 superfamily and were full length, with intact ORF2s, but contained premature stop codons in ORF1 of the consensus elements. Three non-autonomous piggyBac elements were recovered and verified via their TTAA target site duplications. Finally, 22 elements in the TcMariner superfamily were identified including three potentially autonomous elements. BLASTp results from ORFs in these transposons revealed similar domain organization in each. ORFs ranged in length from 493 to 594 amino acids and two of the three contained a helix-turn-helix, Tc5 transposase, and DDE-like integrase domain while the third lacked the initial helix-turn-helix domain. All TcMariner elements had terminal inverted repeats of 12-26 bps that began with CAG and TA target site duplications.

HelitronScanner was used to identify low copy number *Helitrons* that would have been culled based on the filtering criteria for the RepeatModeler data. As a negative control, searches for *Helitrons* were run in parallel on *M. natalensis*, and a shuffled version of the *M. natalensis* genome. HelitronScanner identified 10 elements ranging in size from 2,351 to 14,820 bps in the *M. natalensis* genome and none in the shuffled genome. Several steps were taken to confirm these as true *Helitrons*. First, these elements were used as BLASTn queries against the *M. natalensis* genome to determine copy number. Other than the original locus, no significant hits were found indicating these putative *Helitrons* were single copy. Next, we used BLAT to compare the putative *Helitrons* to the *Myotis lucifugus* genome. In nine of the 10 cases, full-length elements were found, but none overlapped with known *Myotis lucifugus Helitrons*, in the tenth case, no homologous sequence was found in *Myotis lucifugus*. Next, putative *Helitrons* were compared to all known TEs in RepBase. The putative *Helitrons* identified by HelitronScanner lacked homology to other known *Helitrons*. Finally, ORFs were identified with ORF Finder. The largest ORF from each putative *Helitron* was used as a BLASTp query. None of these searches identified domains associated with *Helitrons* (ex. Zinc-finger domains, replicase, helicase, etc. [28]) and a majority failed to recover significant hits to any known protein. Based on these results, the sequences recovered by HelitronScanner are likely artifacts of the search methodology and not true *Helitrons,* since these loci are single-copy, present in the *Myotis lucifugus* genome, lack homology to other known *Helitrons*, and lack ORFs expected in *Helitrons*.

To identify lineage-specific elements, the validated TEs were compared to all known genomes in the NCBI genomes database and classified as lineage-specific, ancestral, or disjunct based on the 50 best BLASTn hits. In all, six elements were specific to *M. natalensis*, five solo LTRs and one non-autonomous piggyBac. Seventeen of the validated elements were found in other vespertilionid bats, including eight transposons in the TcMariner superfamily. The best BLASTn hits for seven elements were to non-chiropteran taxa. Of these, six were cut and paste transposons (5 Tiggers and 1 piggyBac) and one was a LTR. All five Tiggers are elements previously identified in other non-chiropteran taxa and thus represent ancient transposons. One element, Tigger1_MNa shared similarity to more than twenty insertions in the brown kiwi (*Apteryx australis*) genome. All hits were ≥ 97 % similar across ≥ 92 % of the entire *M. natalensis* Tigger1_Mna element. Since our de novo analysis only masked chiropteran-specific elements, these elements, known from other non-chiropteran taxa, were not identified in the initial masking procedures. The closest BLASTn hit to the remaining nine elements was to *Pteropus alecto*, a pteropodid bat. The pteropodid bats are only distantly related to *Miniopterus* among bats and some elements likely represent subfamilies diverged from TEs in the ancestral bat genome. These elements were re-classified as “ancestral”.

**Fig. 1:**
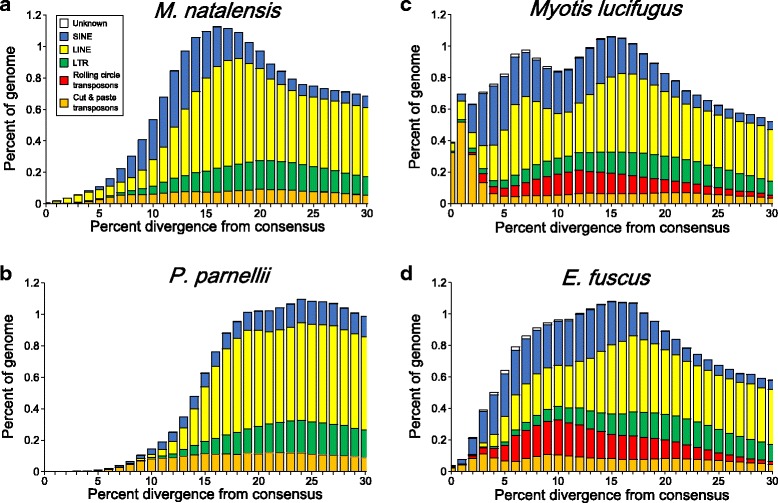
Transposable element accumulation profiles in **a** *Miniopterus natalensis*, **b** *Pteronotus parnellii*, **c** *Myotis lucifugus*, and **d** *Eptesicus fuscus*. Kimura 2-parameter genetic distances were calculated between each repeat in the genome and the putative consensus for its subfamily. Distance values were binned based on transposable element type to visualize the accumulation of transposable elements over time. Due to their high mutation rate, CpG sites were excluded from genetic distance calculations

Individual TE insertions in the *M. natalensis* genome were annotated using the final validated TE library that was combined with all known mammalian repeats in RepBase. For comparison, *Myotis lucifugus*, *E. fuscus*, and *P. parnellii* were processed alongside *M. natalensis*. All four bat genomes contained similar quantities of TEs ranging from 24-27.5 % (Table 1). Class II content was more variable between species than any of the retrotransposon categories. Cut and paste transposons comprised only 1.52 % of the *M. natalensis* genome and less than 0.01 % was derived from *Helitrons*. In general, DNA transposon content in *M. natalensis* was more similar to the outgroup, *P. parnellii*, than to the vespertilionids (Table 1). The repeat accumulation profile for *M. natalensis* (Fig. 1a) indicates that a significant majority of Class II elements are heavily mutated when compared to the presumed ancestral sequence, indicating long periods of inactivity within the genome. In fact, TEs in the *M. natalensis* genome appear to be accumulating less rapidly than in the past. *M. natalensis* and *P. parnellii* (Fig. 1b) both show declining accumulation of Class II elements and negligible *Helitron* content. Both vespertilionid bats show appreciable levels of *Helitron* content and recent accumulation of cut and paste elements (Fig. 1c and d).

**Table 1:** Transposable element content. The number of bases and percent of the genome derived from transposable elements was calculated in four species of bats. The percentage of the genome occupied by transposable elements was calculated based on the total genome size, excluding ambiguous regions or scaffold gaps ("N"s)

Classification	*Miniopterus natalensis*		*Myotis lucifugus*		*E. fuscus*		*P. parnelli*	
	Bases	Percentage	Bases	Percentage	Bases	Percentage	Bases	Percentage
Transposable elements	415,627,321	23.95 %	518,680,444	27.50 %	478,933,702	26.58 %	383,285,246	24.76 %
Class I Retrotransposons	388,593,157	22.39 %	424,243,455	22.50 %	383,040,593	21.26 %	346,459,100	22.37 %
Long Terminal Repeats	69,316,646	4.00 %	72,931,404	3.88 %	71,532,426	3.97 %	68,573,030	4.43 %
ERV	1,092,720	0.06 %	1,038,965	0.06 %	1,149,410	0.06 %	1,499,592	0.10 %
ERV1	23,526,841	1.36 %	28,053,951	1.49 %	26,324,280	1.46 %	19,669,702	1.27 %
ERV2	431,937	0.02 %	7,857,605	0.42 %	4,951,316	0.27 %	391,300	0.03 %
ERV3	41,929,575	2.42 %	34,495,447	1.83 %	37,663,204	2.09 %	45,513,437	2.94 %
Gypsy	357,161	0.02 %	220,101	0.01 %	272,666	0.02 %	600,987	0.04 %
LTR	1,978,412	0.12 %	1,265,335	0.07 %	1,171,550	0.07 %	898,012	0.05 %
Long INterspersed Elements	241,612,217	13.92 %	242,431,627	12.85 %	210,106,281	11.66 %	225,554,475	14.57 %
L1	240,801,801	13.88 %	241,785,916	12.82 %	209,396,239	11.63 %	224,541,665	14.51 %
L2	63,018	0.00 %	42,706	0.00 %	53,584	0.00 %	158,866	0.01 %
Penelope	3,390	0.00 %	2,619	0.00 %	1,994	0.00 %	3,371	0.00 %
R4	24,859	0.00 %	14,178	0.00 %	21,390	0.00 %	35,098	0.00 %
RTE	467,222	0.03 %	425,242	0.02 %	428,374	0.02 %	431,357	0.03 %
RTEX	246,560	0.01 %	156,754	0.01 %	198,740	0.01 %	376,073	0.02 %
Tx1	5,367	0.00 %	4,212	0.00 %	5,960	0.00 %	8,045	0.00 %
Short INterspersed Elements	77,664,294	4.47 %	108,880,424	5.77 %	101,401,886	5.63 %	52,331,595	3.37 %
Unclassified	141,822	0.01 %	113,971	0.01 %	119,602	0.01 %	197,376	0.01 %
tRNA	77,501,269	4.46 %	108,745,685	5.76 %	101,255,190	5.62 %	52,075,143	3.36 %
7SL	1,048	0.00 %	1,775	0.00 %	1,914	0.00 %	3,786	0.00 %
5S	20,155	0.00 %	18,993	0.00 %	25,180	0.00 %	55,290	0.00 %
Class II DNA transposons	26,535,664	1.53 %	91,629,080	4.85 %	92,568,583	5.14 %	36,073,984	2.34 %
Cut and Paste	26,433,314	1.52 %	47,434,627	2.51 %	35,693,046	1.98 %	35,940,177	2.33 %
Kolobok	10,135	0.00 %	8,065	0.00 %	10,145	0.00 %	16,113	0.00 %
MuDR	13,048	0.00 %	12,651	0.00 %	13,221	0.00 %	21,955	0.00 %
PiggyBac	366,671	0.02 %	261,766	0.01 %	941,162	0.05 %	117,137	0.01 %
TcMar-Mariner	7,537,182	0.43 %	7,941,486	0.42 %	10,197,575	0.57 %	11,885,374	0.77 %
hAT	18,506,278	1.07 %	39,210,659	2.08 %	24,530,943	1.36 %	23,899,598	1.55 %
Rolling circle	102,350	0.01 %	44,194,453	2.34 %	56,875,537	3.16 %	133,807	0.01 %
Helitrons	102,350	0.01 %	44,194,453	2.34 %	56,875,537	3.16 %	133,807	0.01 %
Unknown	498,500	0.03 %	2,807,909	0.15 %	3,324,526	0.18 %	752,162	0.05 %

In addition to accumulation profiles, average genetic distances between consensus elements and TE loci were calculated for all DNA transposons that occupied more than 10 Kb in any of the bat genomes examined (Additional file 1: Table S1). In all, 248 different DNA transposons met these criteria. A portion of this data is presented in Table 2. Genetic distances can be used as a relative metric for age and combined with presence or absence in other species to understand TE dynamics within this group. The most common superfamily of TEs in the genomes examined were hATs (133 of 248 elements). Generally, hATs fell into two categories; 1) they were present in some combination of vespertilionid genomes or, 2) they were found in *M. natalensis* and also identified in the vesper bats and the outgroup, *P. parnellii*. Based on genetic distance, nhAT-100_EF was the most recent hAT transposon in *M. natalensis* (Table 2). This transposons, nhAT-100_EF, was present in all four taxa examined and genetic distances fell within a limited range (18.69-19.75 %). *Helitrons* followed a similar pattern to hATs; the two *Helitrons* identified in *M. natalensis* were shared by all three other species and heavily mutated (Table 2). TcMariner transposons, in general, were shared among all analyzed taxa, with a limited number of exceptions. Two elements were not identified in *P. parnellii.* Four older elements with average genetic distances greater than 23 % were found only in *P. parnellii*. Interestingly this analysis, which relied on RepeatMasker searches, identified a single element restricted to *M. natalensis* (nTIGGER-7_MNa). The BLAST searches used to identify lineage-specific repeats (described above) identified a single homologous sequence in another bat, *Rhinolophus ferrumequinum* (99 % query coverage, 85 % identity, 2e-57 E value), but not to other vesper bats. It is possible that this element is specific to *M. natalensis* since it was only found at one locus in one other species. In either case, since *R. ferrumeguinum* was not in our RepeatMasker searches, the distribution of this element among the taxa examined appears reasonable. Finally, most unclassified DNA transposons, Kolobok, and MuDRs were ancestral elements with high genetic diversity and present in all four taxa.

**Table 2:** The average Kimura 2-parameter, genetic distance was calculated among all insertions for each element. Highly mutable CpG sites were excluded from distance calculations

		Within group genetic distance (average)			
Super Family	Element	*Miniopterus natalensis*	*Myotis lucifugus*	*Eptesicus fuscus*	*Pteronotus parnellii*
hAT	nhAT-100_EF	18.69	18.94	19.16	19.75
Helitron	Helitron1Nb_Mam	29.66	30.09	30.45	30.5
Helitron	Helitron3Na_Mam	32.74	32.87	33.57	33.93
PiggyBac	nPiggyBac-2_MNa	1.35	NA	NA	NA
PiggyBac	piggyBac2b_Mm	NA	1.91	NA	NA
PiggyBac	nPiggyBac-1_MNa	2.03	NA	NA	NA
PiggyBac	piggyBac1_Mm	NA	NA	2.18	NA
PiggyBac	npiggyBac-2_EF	NA	NA	2.86	NA
PiggyBac	npiggyBac-1_EF	NA	4.16	4.77	NA
PiggyBac	npiggy1_Mm	NA	NA	5.3	NA
PiggyBac	piggyBac_2a_Mm	NA	6.78	NA	NA
PiggyBac	nPiggyBac-3_MNa	7.58	NA	NA	NA
PiggyBac	piggyBac2_Mm	8.16	12.35	38.09	NA
TcMariner	nTIGGER-7_MNa	8.08	NA	NA	NA
TcMariner	nTIGGER-12_MNa	8.98	9.54	9.76	NA
TcMariner	nTIGGER-18_MNa	9.99	9.9	10.33	NA
TcMariner	TIGGER-1_Mna	13.63	14.41	14.6	15.66
TcMariner	TIGGER1	14	14.31	14.84	15.96

Distances were only calculated if the element occupied more than 10 kilobases in a genome. For species were elements were absent or occupied less than 10 kilobases of their genome, values are given as "NA"s. A limited number of transposons are shown here. A complete table displaying the average genetic distances of all elements is provided as Additional file 1: Table S1

## Discussion

Active DNA transposons are rare in mammals. To date, only the vespertilionid family of bats are known to have significant levels of active Class II elements. *Miniopterus* is the sole genus of the recently elevated family Miniopteridae, the sister family to Vespertilionidae [9]. Previous studies indicated that *Miniopterus* lacks the *Helitrons* found in vesper bats and may harbor limited piggyBac activity [6, 8]. Based on these results, it has been assumed that the horizontal transfer of DNA transposons occurred in an ancestral vespertilionid bat subsequent to the divergence of *Miniopterus*. Complete analysis of the *M. natalensis* genome generally supports previous conclusions with slight modifications, namely that limited Class II accumulation of TcMariner and piggyback elements indicate their presence in the *Miniopterus* + vespertilionid ancestor. It is possible that biases introduced with sequencing chemistries, genome assembly methods, and bioinformatics analyses negatively influence the recognition of repetitive sequences. Highly repetitive sequences with low nucleotide diversity represent a significant problem for genome assembly methods. In addition, the culling of very low copy number elements (n = <10) from the initial de novo repeat identification with RepeatModeler could bias estimations slightly downward. While these influences are expected to be minimal, they cannot be accurately quantified and all results should be interpreted with these caveats in mind.

### Species distribution of TEs identified in M. natalensis

De novo identification of TEs and manual curation identified several elements that are novel or exhibit interesting taxonomic distributions. Tigger1_MNa shared homology with twenty insertions in the brown kiwi genome and is closely associated with the consensus sequence for TIGGER1 originally identified in the human genome. These two consensus elements (Tigger1_MNa and TIGGER1) share almost 97.5 % similarity despite individual insertions being heavily mutated in the respective genomes [29] (Table 2). To demonstrate horizontal transfer between *M. natalensis* and the brown kiwi, an element must have a disjunct phylogenetic distribution and high sequence similarity in multiple species beyond what is expected based on divergence times [30]. The BLASTn results for Tigger1_MNa seem to support a disjunct distribution, but its heavy mutation load may be within expectations based on a neutral mutation rate and the respective divergence times of these taxa [30]. Other factors giving the appearance of a disjunct species distribution, such as sequence contamination in the kiwi genome, cannot be conclusively ruled out.

BLAST searches identified several elements specific to the *M. natalensis* genome indicating their emergence sometime in the last 37.5 [31] to 43 my [9]. Five of these are LTRs but one non-autonomous piggyBac DNA transposon (npiggyBac-3_Mna) is specific to *M. natalensis* based on comparisons to all currently available genomes. npiggyBac-3_Mna was present in the *M. natalensis* genome at low frequency (577 copies). In addition to npiggyBac-3_Mna, previous work noted that a small region associated with *Myotis lucifugus* piggyBac2_ML (bp 1,536-2,340) was also present in *Miniopterus* sp. [6]. Analysis of the entire *M. natalensis* genome indicates that the piggyBac2_ML fragment amplified by Ray et al. [6] is present in the *M. natalensis* genome as part of the larger piggyBac2_Mm element. RepBase does not recognize piggyBac2_ML (accessed 1 April 2016). Instead, it contains piggyBac2_Mm, the *Microcebus murinus* counterpart to piggyBac2_ML that is presumed to have been horizontally transferred between *Microcebus murinus* and *Myotis lucifugus* [32]. To be consistent with RepBase naming conventions, we refer to piggyBac2_ML from Ray et al. [6] as piggyBac2_Mm. In all, RepeatMasker identified fewer than 80 piggyBac2_Mm loci occupying 58,499 bps in the *M. natalensis* genome.

**Fig. 2:**
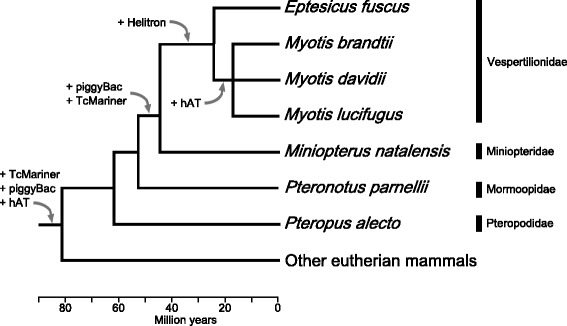
A simplified tree depicting relationships among specimens examined. Time since divergence for each species is Time Tree of Life divergence estimate [39] from timetree.org. Relationships within *Myotis* are unresolved due to conflicting mitochondrial and nuclear phylogenies [40]. The gain of relevant, active transposons are plotted on respective nodes

These results suggest that the PCR-based analyses of Ray et al. [6] were accurate in their identification of piggyBac2_Mm distribution among chiropterans. In that work, however, piggyBac2_Mm was absent in non-*Myotis* vesper bats. RepeatMasker results identify piggyBac2_Mm in *E. fuscus*, but in a heavily mutated and truncated form (Table 2) implying that piggyBac2_Mm elements in *E. fuscus* are ancestral elements misidentified as piggyBac2_Mm. The presence of closely related piggyBacs in *Myotis lucifugus* and *M. natalensis* could be explained by two possible scenarios: horizontal transfer of piggyBac2_Mm between *M. natalensis* and a *Myotis* sp. or invasion of piggyBac2_ML into the *Miniopterus* + vespertilionid ancestral genome, and subsequent loss in the lineage leading to *Eptesicus*. The genus *Myotis* occupies a basal clade within Vespertilionidae [33] meaning that if piggyBac2_Mm was present as a single or few copies, a single loss could explain the presence of piggyBac2_Mm in *Myotis* and *M. natalensis*, but not *Eptesicus*. Further supporting this scenario, piggyBac2_Mm contains more genetic diversity (8.16-12.35 %; Table 2) than other piggyBac elements that are limited to single species. It is likely that piggyBac2_Mm is an older subfamily of elements and may even be one of the first transposons to invade the bat genomes. On the other hand, horizontal transfer of piggyBac2_Mm involving *Myotis lucifugus* and *Microcebus murinus* (the mouse lemur) has been reported previously [32]. The distribution of these three genera (*Microcebus*, *Miniopterus*, and *Myotis*) all include portions of Africa and/or Madagascar, which allows for the possibility of such transfers in ancestral species (assuming similar ancestral distributions). Based on the current data, piggyBac2_Mm likely represents an invasion in an ancestral bat genome followed by a loss in *E. fuscus* (Fig. 2). In either case, *M. natalensis*, *Myotis lucifugus*, and *E. fuscus* each have lineage-specific, highly similar piggyBac transposons indicating some level of transposition in these genomes (Table 2).

### TE invasions in Vespertilionidae and Miniopteridae

Just under 30 % of the *M. natalensis* genome is derived from TEs. Though there is evidence of lineage-specific accumulation, the *M. natalensis* genome appears to have experienced minimal DNA transposon activity when compared to vespertilionids (Fig. 1). Around 26.5 Mb (1.5 %) of the genome is derived from Class II elements compared to ~5 % in the vespertilionid bats (Table 1). The bulk of these DNA transposon in *M. natalensis* are cut and paste DNA transposons, specifically hATs which account for 70 % of all transposon content. Several observations indicate the hAT elements were deposited in a distantly related ancestor of these taxa. First, analysis of transposons in primate genomes identified significant transposon activity from TcMariners, piggyBacs, and hATs during the eutherian radiation 81-150 mya, hATs being the dominant transposon [34]. Second, the most abundant transposon in *M. natalensis*, hATs, were highly mutated and present in all the Vespertilionidae, *M. natalensis* and *P. parnellii*; indicating ancestral accumulation (Table 2). Third, the quantity of cut and paste transposons in *M. natalensis* is more similar to *P. parnellii* than its more closely related vesper relatives. Fourth, the methods used herein were capable of finding lineage-specific elements yet only one new piggyBac was identified (compared to five LTRs). Based on these results, it seems clear that the bulk of cut and paste DNA transposons were deposited prior to the Chiropteran divergence meaning that at least 70 % of all DNA transposon activity in *M. natalensis* is ancestral.

*Helitrons* are not as common as cut and paste transposons in the *M. natalensis* genome, occupying less than 100 Kb. Two *Helitrons* (Helitron1Nb_Mam and Helitron3Na_Mam; Table 2) appear to have been active prior to the emergence of Chiroptera based on their presence in the taxa examined. HelitronScanner, failed to identify Helitron1Nb_Mam and Helitron3Na_Mam, likely due to the high mutation load they carry (>30 % on average; Table 2). The failure to identify novel *Helitrons* through structural searches and the low copy numbers of ancestral *Helitrons* identified via homology makes it reasonable to conclude that the *Helitrons* invasion into the vesper bats occurred subsequent to their divergence from *Miniopterus*.

The lack of significant cut and paste transposon accumulation and the absence of *Helitrons,* allows us to place more precise taxonomic and temporal limits on the DNA transposon invasion of an ancestral bat genome (Fig. 2). The presence of a limited number of TcMariner and piggyBac families present in *M. natalensis* and the vespertilionids seems to indicate that the acquisition of DNA transposons began just before the divergence of *Miniopterus* and the vespertilionids. *Helitrons* and hATs were introduced into an ancestral vespertilionid subsequent to the divergence of *Miniopterus*. Lineage-specific cut and paste DNA transposons reached much higher copy numbers in the vespertilionids genomes (Fig. 1c and d) than in the *M. natalensis* genome (Fig. 1a).

## Conclusions

The results presented here confirm and expand upon previous findings regarding the distribution of DNA transposons in bats [3–6] and suggest several avenues of research. For example, if an ancestral *Miniopterus* + vespertilionid bat was exposed to DNA transposons, what factors were responsible for the differential accumulation in the daughter lineages? How have genomic defense mechanisms against TEs evolved in presence/absence of DNA transposons [35]? What vectors are responsible for transferring Class II elements to these bats [36]? Finally, what role do TEs play in the generation of taxonomic and genomic diversity? The rapid diversification of the vespertilionid bats is temporally associated with the acquisition of DNA transposons [7]. Individual TE insertions are generally neutral or deleterious, but instances of exaptation are known (reviewed in [37]). Beyond individual TE insertions, TE activity in general may be beneficial, allowing species to rapidly adapt to changing environments more quickly than relying on point mutations alone [38]. *Miniopterus* and the vespertilionids may represent extremes in the possible diversity of mammalian TE repertoires in sister taxa. By taking advantage of these contrasting compositions, it may be possible to answer specific questions regarding TEs and their role in genome evolution.

## Abbreviations

TEs, LINE, SINE, LTR, mya, ORFs, Kb

## Additional files

Additional file 1: The average Kimura 2-parameter, genetic distance was calculated among all insertions for each element. Highly mutable CpG sites were excluded from distance calculations. Distances were only calculated if the element was occupied more than 10 kilobases in a species. For species where elements were absent or occupied less than 10 kilobases of their genome, values are given as "NA"s. Calculations for all meeting these critera are shown here. Table 2 in the manuscript is reduced fascimilie of this table containing only elements specifically discussed in the text. (XLSX 22 kb) Additional file 2: Novel *Miniopterus natalensis *transpsoable elements were identified using de novo methods and manual curation. Sequences are in FastA format. (FAS 39 kb)
